# Single-incision laparoscopic intraperitoneal onlay mesh hernioplasty for anterior abdominal wall hernia: A safe and feasible approach

**DOI:** 10.4103/0972-9941.72377

**Published:** 2011

**Authors:** Prabal Roy, Anushtup De

**Affiliations:** Department of General and Minimally Invasive Surgery. Asian Institute of Medical Sciences, Faridabad, Haryana, India

**Keywords:** Laparoscopic intraperitoneal onlay mesh hernioplasty, laparoscopic ventral hernia repair, single-incision laparoscopic surgery

## Abstract

**BACKGROUND:**

Single-incision laparoscopic surgery is being increasingly performed in experienced laparoscopic centres. The primary aim is at improving the cosmetic outcome of surgery without compromising the safety of the operation. The challenge of this surgery lies in limited range of movement of the instruments due to proximity of working ports and limited triangulation.

**METHODS:**

We describe our first four consecutive cases of single-incision laparoscopic intraperitoneal onlay mesh hernioplasty for anterior abdominal wall hernia repair during a period of June to July 2009. Operative time, hospital stay and postoperative pain were assessed, and follow up was done for 3 months.

**RESULTS:**

Three patients were discharged on first postoperative day and one on second postoperative day without any complications.

**CONCLUSIONS:**

Based on our experience, we believe that the procedure is feasible without additional risk. Cosmetic benefit is clear; however, beyond that the actual outcome with respect to patient recovery, postoperative pain and long-term complications needs to be evaluated and compared to standard laparoscopic intraperitoneal onlay mesh hernioplasty.

## INTRODUCTION

Single-incision laparoscopic surgery has recently emerged as a possible alternative to conventional laparoscopic surgery in a variety of surgeries.[1–4] It is being developed with the aim of decreasing the invasiveness of traditional laparoscopy and improving cosmetic outcome.

Single-incision laparoscopic cholecystectomy was first reported by Navarra *et al*.[1] in 1997 and single-incision laparoscopic appendicectomy by Esposito[2] in 1998. Subsequently, the approach was applied in urology[3] in 2005 and obesity surgery[4] in 2008. The transient break can possibly be explained by the lack of technical support which has remained unavailable until recently. Nowadays, special multilumen ports that allow simultaneous instrument placement[5] and articulating and bent instruments as well as adjustable laparoscopes have become available. However, the use of specialised ports and articulating instruments considerably increases the costs of surgery. Keeping this aspect in consideration, we have been using conventional laparoscopic instruments in single-incision surgery with minimal additional technical difficulty, comparable surgical outcome and good patient satisfaction.

In procedures such as hernia repair where laparoscopic approach still remains a matter of debate, single-incision laparoscopic surgery may prove to be one of the points in its favour. However, in spite of the obvious theoretical benefits, the practical benefits and potential risks remain unclear. We report our technique and operative difficulties of single-incision surgery for ventral hernia in four consecutive cases at our centre.

## METHODS

### Patient Profile

Four patients presenting with history of swelling over abdomen of an average duration of 1 year 10 months (range 6 months–3 years) and clinically confirmed anterior abdominal wall hernia were given the option to undergo single-incision laparoscopic intraperitoneal onlay mesh hernioplasty. They consented to the technique after all the risks and benefits were properly explained. All operations were performed by the same surgical team that had already performed over 50 single-incision laparoscopic cholecystectomies. Three of the patients were males and the fourth one a female. The average age was 43.75 years (range 30–55 years). Two patients had associated comorbidities of hypertension. Two of the patients had incisional hernia in their middle and lower abdomen following exporatory laparotomy and open abdominal hysterectomy, respectively. The other two had paraumbilical defect. Two of the patients had an irreducible hernia. Postoperatively, the patients were questioned on their expectations from the surgery in terms of postoperative pain and scar related outcome.

### Operative Technique

**Figure 1:**
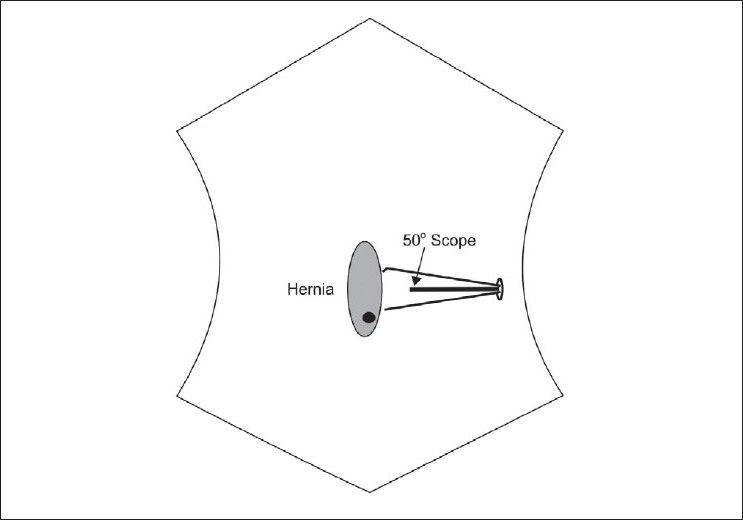
Graphic representation of port placement in single-incision intraperitoneal mesh hernioplasty

Pneuperitoneum was established using a veress needle placed at left hypochondrium (Palmers point). Intra abdominal pressure was maintained at 12 mm Hg. A 1.5-2 cm linear incision was made at the left flank and a 10-mm port was placed. One or two 5 mm trocars were placed through the same incision, about 0.5 cm anteriorly on either side of the 10 mm port [Figure 1].

**Figure 2:**
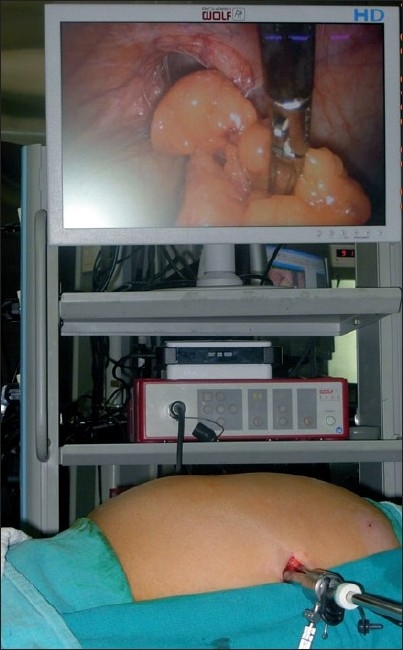
Single-incision port placement with omentum reduced from hernial defect

The surgical technique is the same as for standard laparoscopic intraperitoneal onlay mesh hernioplasty. Omental contents was reduced [Figure 2]. The hernia defect was closed by extracorporeal continuous no. 1 nylon sutures and intraperitoneal pressure was decreased to 10 mm Hg. Then, a dual mesh was placed which was held with four transfascial sutures passed with a hernia needle and additionally supported with tackers (5 mm ProTack Auto Suture^™^). Local infiltration of 2% Lignocaine with 0.5% Bupivacaine was given at the incision and transfascial suture placement sites.

## RESULTS

Two patients had irreducible omentum as the content in the hernia sac and reduction was achieved by external pressure and intra abdominal manipulation without any intraoperative difficulties. The fascial defect was routinely closed with no. 1 nylon sutures. The mean operative time was 38.75 min (range 30–50 min). There were no intraoperative complications. Postoperative analgesia was covered adequately by inj. Diclofenac sodium 75 mg twice daily on the day of surgery. No patient required added analgesia. Three patients were discharged on the next day of surgery. One patient was discharged on the second postoperative day due to transient pain-related issues. Patient satisfaction was assessed based on a questionnaire at follow up in out-patient department after 7 days and lesser pain than expected was reported by three patients. All four patients were satisfied at the cosmetic outcome after 3 months. Recovery was uneventful on a follow up of 3 months. None of the patients had wound-related complications including seromas.

## DISCUSSION

The popularity of laparoscopic ventral hernia repair is increasing.[6] The laparoscopic approach offers several key advantages over the open approach, including low risks of infection and shortened hospital stay in addition to reduction in complication rates and postoperative ileus. One major advantage of the laparoscopic approach is that it provides wide mesh overlap in an underlay position similar to the open retro-rectus repair, without the associated soft tissue dissection. Additionally, the laparoscopic approach provides a unique internal view of the entire abdominal wall, enabling the surgeon to identify remote small swiss cheese-type defects. Single-incision laparoscopic intraperitoneal onlay mesh hernioplasty performed at our institution is principally the same as the conventional laparoscopic procedure in that conventional laparoscopic instruments are used in it even though it is performed through a single-incision.

One of the most controversial areas of laparoscopic ventral hernia repair is the appropriate method of mesh fixation. Several experienced laparoscopic ventral hernia surgeons have advocated the routine use of transfascial fixation sutures.[7,8] We have routinely used a minimum of four transfascial sutures reinforced with tackers in standard laparoscopic intraperitoneal mesh hernioplasty and have maintained the same operative technique in single-incision procedure.

On extensive Medline search, we have observed that single-incision intraperitoneal onlay mesh hernioplasty has not yet been reported in literature. Single-incision surgery is technically more challenging compared with conventional laparoscopic surgery. In-line positioning of the laparoscope, close proximity of working instruments with limited triangulation, limited range of motion of the laparoscope and instruments, and decreased number of ports all contributed to increased difficulty. However, with expertise and experience it is possible to overcome the difficulties and perform the surgery within the limitations of safety.

It might be noted that the hernias repaired were relatively small in size with the largest defect measuring 7 × 6 cm and two of them being an incisional hernia resulting in a swiss cheese defect. The rest of the hernias were average 5 × 4 cm paraumbilical hernias. Also, none of the hernias had bowel as a content of the hernia sac. Nevertheless, providing all the advantages of laparoscopic hernia repair within the limits of operative safety, we find single-incision laparoscopic intraperitoneal mesh hernioplasty both safe and feasible. With further technological advancement and as we gain experience, we hope that possibly larger defects can be managed by this novel technique.

One potential disadvantage we noted was that the size of the incision in the skin was slightly longer (1.5 - 2 cm) than the standard incision at 10 mm port in laparoscopic surgery and also the placement of one/two 5 mm ports in relative proximity. This raises the question of port site hernia postoperatively. However, we routinely close the muscle layer as in case of all 10 mm ports in standard laparoscopic surgery in which port site hernias are extremely uncommon. So, we do not anticipate any incisional hernia rate moving forwards in our technique.

A variety of single-incision surgical procedures have been described in recent times. Apart from the cosmetic benefit which is obvious, the other proposed benefits include less incisional pain and the freedom to convert to standard laparoscopy or open procedure without any additional morbidity. Nonetheless, in the present scenario, single-incision laparoscopic onlay mesh hernioplasty for anterior abdominal wall hernia is both safe and feasible.
